# A machine learning application for raising WASH awareness in the times of COVID-19 pandemic

**DOI:** 10.1038/s41598-021-03869-6

**Published:** 2022-01-17

**Authors:** Rohan Pandey, Vaibhav Gautam, Ridam Pal, Harsh Bandhey, Lovedeep Singh Dhingra, Vihaan Misra, Himanshu Sharma, Chirag Jain, Kanav Bhagat, Arushi, Lajjaben Patel, Mudit Agarwal, Samprati Agrawal, Rishabh Jalan, Akshat Wadhwa, Ayush Garg, Yashwin Agrawal, Bhavika Rana, Ponnurangam Kumaraguru, Tavpritesh Sethi

**Affiliations:** 1 Shiv Nadar University, Noida, Uttar Pradesh India; 2 Department of Computational Biology, Indraprastha Institute of Information Technology, Delhi, Okhla Industrial Estate, Phase III, New Delhi, 110020 India; 3 All India Institute of Medical Sciences, New Delhi, India; 4 GL Bajaj Institute of Tech and Management, Greater Noida, Uttar Pradesh India; 5 Netaji Subhas University of Technology, Dwarka, New Delhi, India

**Keywords:** Public health, Computational science

## Abstract

The COVID-19 pandemic has revealed the power of internet disinformation in influencing global health. The deluge of information travels faster than the epidemic itself and is a threat to the health of millions across the globe. Health apps need to leverage machine learning for delivering the right information while constantly learning misinformation trends and deliver these effectively in vernacular languages in order to combat the infodemic at the grassroot levels in the general public. Our application, WashKaro, is a multi-pronged intervention that uses conversational Artificial Intelligence (AI), machine translation, and natural language processing to combat misinformation (NLP). WashKaro uses AI to provide accurate information matched against WHO recommendations and delivered in an understandable format in local languages. The primary aim of this study was to assess the use of neural models for text summarization and machine learning for delivering WHO matched COVID-19 information to mitigate the misinfodemic. The secondary aim of this study was to develop a symptom assessment tool and segmentation insights for improving the delivery of information. A total of 5026 people downloaded the app during the study window; among those, 1545 were actively engaged users. Our study shows that 3.4 times more females engaged with the App in Hindi as compared to males, the relevance of AI-filtered news content doubled within 45 days of continuous machine learning, and the prudence of integrated AI chatbot “Satya” increased thus proving the usefulness of a mHealth platform to mitigate health misinformation. We conclude that a machine learning application delivering bite-sized vernacular audios and conversational AI is a practical approach to mitigate health misinformation.

## Introduction

**Figure 1:**
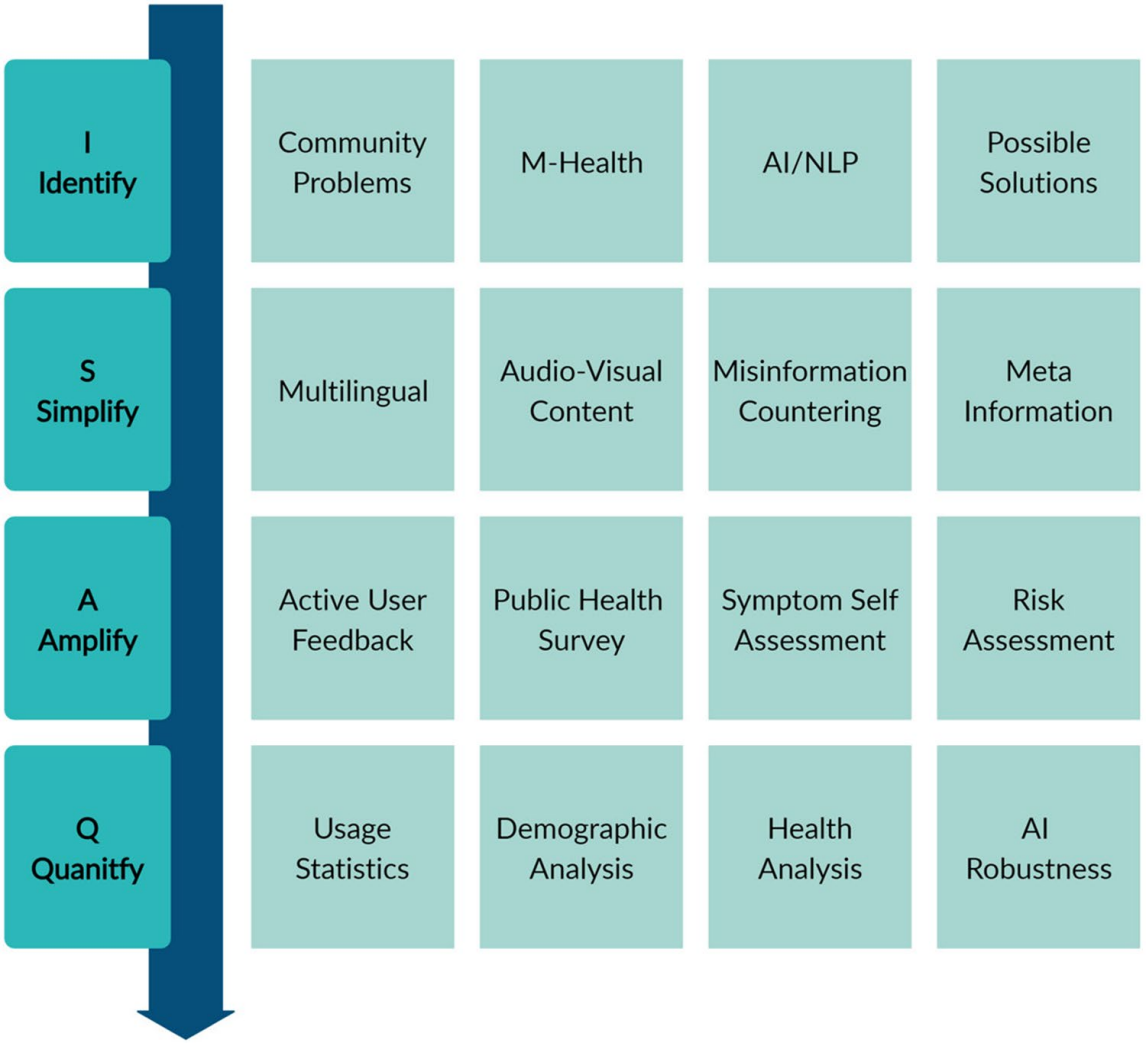
Proposed workflow of the App based upon Identify, Simplify, Amplify and Quantify framework as specified WHO’s EPI-WIN strategy^4^.

Healthcare misinformation is a growing menace in digital societies^1,2^. This is clearly highlighted by the COVID-19 pandemic that has affected over 3.8 million people worldwide, causing a widespread loss in all aspects of daily life^3^. Digital consumption has increased manifolds, creating both an opportunity and a danger in terms of information dissemination. Infodemic has been defined as an overabundance of information, some accurate and some not, making it hard for people to find trustworthy sources and reliable guidance when they need it^4^. The spread of the COVID-19 infodemic was much faster than the pandemic itself and poses a threat to public health^5^. Further, mitigation of misinformation is also vital for raising correct awareness for the primary prevention of most communicable and non-communicable diseases. Mobile health (mHealth), coupled with verified health information, can serve as an information dispensing tool to tackle the spread of misinformation. Clear and effective communication of preventive measures and updated information is essential. To achieve this goal, designing a trustworthy app that helps navigate the information deluge can be crucial. Therefore, recognizing the potential of mHealth platforms, we developed WashKaro, a multi-pronged AI approach for Infodemic Management. WashKaro was driven by the imminent need to raise Water, Sanitation, and Hygiene (WASH) awareness and combines English (WASH) with vernacular (Karo, meaning "Do" in Hindi) for mitigating the spread of COVID-19. OnAir is a feature on the WashKaro app which combines Natural Language Processing (NLP) to match news articles with WHO guidelines. Conversational AI (Satya, meaning "Truth" in Hindi) reaches out to the community as audio-visual content in local languages. To keep the information relevant, WashKaro provides daily news matched with WHO guidelines^6^, WHO directive-based Symptom Self-Assessment tool, and human-vetted information delivering these in Hindi, the most widely understood local language across India. Since India is one of the largest and fastest-growing markets for digital consumers, with 560 million Internet subscribers in 2018^7^, and about 60% using mHealth technologies^8^, this offered a unique opportunity to test WashKaro. The study is based on the WHO’s Information Network for Epidemics (EPI-WIN)^9^ strategy, covering four strategic areas of work to tackle the infodemic, as shown in Fig. 1.

Prevention of disease using interventions of Artificial Intelligence, Machine learning, and NLP has been a significant breakthrough in the era of Covid-19. There has been a rampant spread of misinformation related to oxygen, availability of oxygen beds, vaccines, drugs, and many other things. For authentication and validation of such information web search engines have been created^10^, various IoT and AI-based tools have been created to raise awareness for concerns related to handwashing, social hygiene, maintaining social distancing, and wearing masks^11,12^.

The methodology used for this study is centered around the WHO EPI-WIN strategy, which covers four strategic areas of work to respond to the infodemic. The first area of coverage is focused on identifying the problem at hand, given the current evidence and information to promote and form public policies strategically. We have identified the context-specific community problems and the potential of mHealth, AI, and NLP in order to acknowledge possible solutions. This is followed by simplifying the enormous amount of information currently available across multiple sources to disseminate accurate information in a simplified manner. In order to achieve this objective, multilingual support is provided in the form of audio visual-based content. The spread of misinformation is tackled by providing information such as Mythbusters and government updates along with meta-information in the form of geographic coordinates of essential facilities. We also offer periodic hand washing reminders. In case of such an unforeseen event, it is vital to amplify the intervention by means of establishing two-way communication with the intended audience to tailor the advice and messages. This has been catered to by engaging users in active feedback based involvement, participating in enhancing the AI proposed model along with any generic feedback in an audio format. A public health survey and symptom self-assessment are crucial components in amplifying our study. In order to devise constantly evolving strategies, it is essential to validate the methodology and quantify the infodemic. WashKaro application statistics, demographic analysis of public health surveys, health analysis of at-risk population using symptom self-assessment, and user agreement on AI-based intervention is critical to quantify and evaluate.

## Methods

WashKaro was developed as a holistic mHealth solution that could serve as a one-stop AI-powered infodemic management suite during the current COVID 19 pandemic. The underlying strategy utilized was Identify-Simplify-Amplify-Quantify, as deployed by the Information Network for Epidemics (EPI-WIN) established by the WHO^4^. The main idea of our application was to provide unsolicited information as bite-sized text and audio in Hindi and English. The mobile application was made available to the general public through Google Play Store and was downloaded by more than 5000 users. We did not select a cohort, and all the responses received from the general public were analyzed to gather real-world evidence about the effectiveness of our machine learning-based messaging intervention. The choice of the Android platform reflects the predominant usage of the platform among smartphone users in India.

The machine learning algorithms helped in filtering correct information from the deluge of news, which were then vetted by medical experts. Gathering raw data from credible sources such as the WHO and consumer-centric daily news articles, we used NLP approaches and Machine Learning (ML) to identify authentic and pertinent information. The information thus extracted was simplified and presented as audio-visual content in Hindi (the most widely understood local language across India), English, and various other vernaculars. By garnering feedback on the relevance of the WHO information provided along with the news pieces, the advice to the individual was tailored according to their personal needs, thus amplifying the reach of appropriate messages. We also offered a WHO directive-based Symptom Self-Assessment tool and numerous categories of human-vetted information in the form of Infographics, MythBusters, geographic information, etc. Forming real-time, on-the-ground, multidisciplinary research partnerships is essential to mitigate the infodemic. Therefore, our entire methodology and infodemic suite is open-source (https://github.com/tavlab-iiitd/WashKaro/tree/master/washkaro-textmatching) and available for the whole of the scientific community to build upon it.

### NLP in healthcare

In the current situation, timely delivery of tenable content to the masses is exceptionally crucial to counter the spread of misinformation. WashKaro targeted this requirement using Natural Language Processing techniques to dispense information sourced through highly trusted WHO outlets such as EPI-WIN, which may not reach the appropriate audience or be too complicated for them^13^.

**Figure 2:**
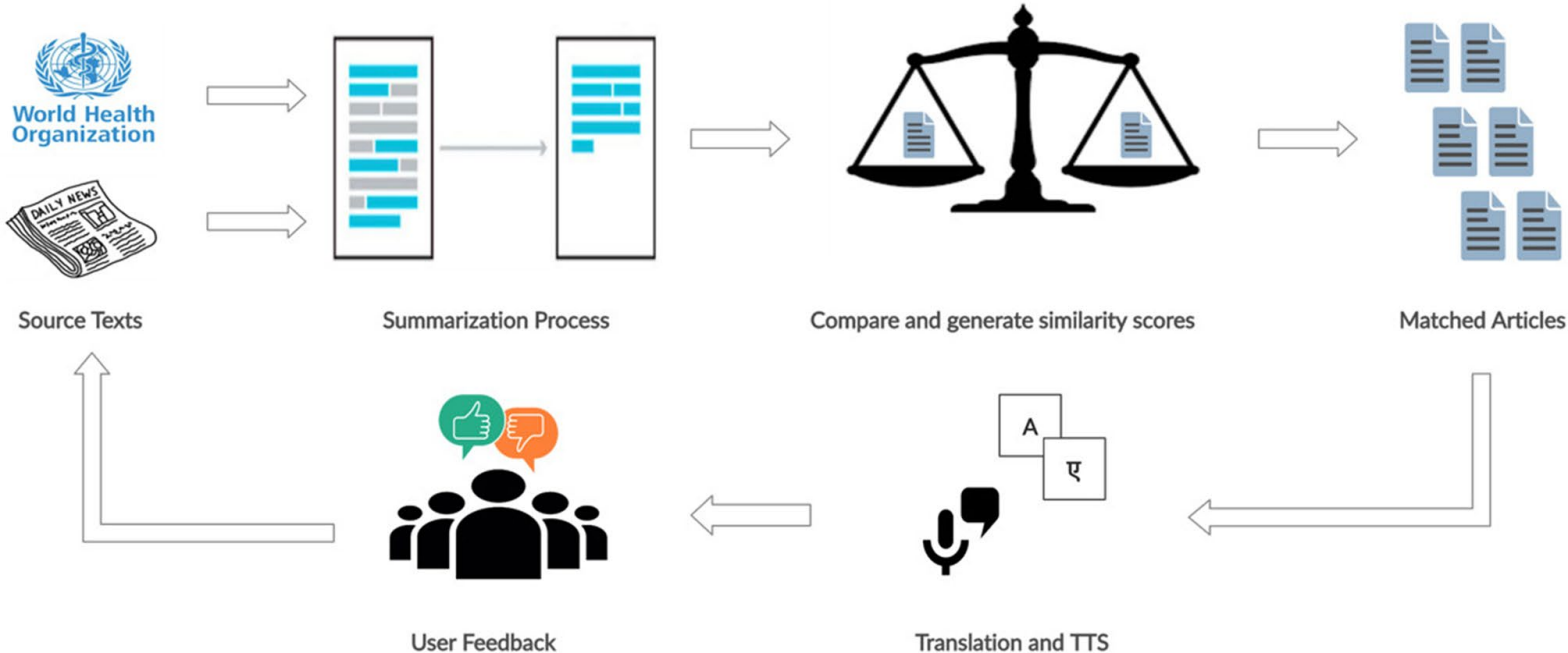
NLP Pipeline. The pipeline takes in news articles and the World Health Organization (WHO) reports and constructs two-level sentence similarity between titles and the full-text to build a similarity score. Finally, the relevant texts are subject to translation and text to speech conversion for local language consumption (Hindi). This figure was made using **Creately** (URL:https://creately.com/).

The NLP pipeline (Fig. 2) involved two datasets: the WHO guidelines and the news articles. Multiple pairs of WHO guidelines and News Articles are generated as an input for the Machine Learning System, extractive ML summarization techniques were used to abbreviate the text. Articles were refreshed on a daily basis (using an automated web scraper) and from the Indian vernacular news source: Dainik Jagran and the WHO website. This data was collated in a csv file which has been used for the modelling task. Pre-Trained Word2Vec Embedding^14^ was used to generate embedding vectors for each word in the two documents. These word vectors were converted to article-level embeddings using Smooth Inverse Frequency^15^. The generated pair of document level vectors are used for the calculation of distance metrics. Cosine similarity was calculated to find the similarity between two embedded vectors. Based on the users’ reviews, the threshold of cosine similarity was set to determine the news articles that will be provided to users subsequently using this AI system^16^. This pipeline served to complement the user's daily news consumption that suits their palette with an appropriate WHO guideline related to COVID-19 and WASH (Water Sanitation and Hygiene), thus augmenting healthcare awareness. In order to enhance engagement and provide increasingly relevant content, user feedback was sought at the end of each matching- the users marked each pair of WHO guideline and news article provided to them as either relevant or irrelevant. This active user feedback aided the machine learning backend in improving with each review by determining the type of news articles the user found relevant to a particular guideline. Further, any new article provided to the user took into account the previous learning, which enabled the deliverance of more relevant information with each feedback cycle.

### Simplification

We made a deliberate attempt to convey context-specific and consumable information in a medium of the user's choice. Infographics based on WHO recommendations were used for effective presentation of preventive measures. Byte sized information packets were delivered in multiple local languages to ensure accessibility to various marginalized groups. Text-to-speech engines helped convert the information to an audio-visual format, thus reaching out to the less educated population. The application made use of the inbuilt Android text-to-speech model available in each android device. Mythbusters and government advisories, critical in countering misinformation and uncertainties surrounding the official guidelines, were credibly sourced and regularly updated. Mythbusters and government advisories, critical in countering misinformation and uncertainties surrounding the official guidelines, were credibly sourced from the official government website of the Ministry of Health and Family Welfare^17^. The guidelines related to COVID-19 present in the MOHFW were updated on a daily basis, and the WashKaro application was simultaneously updated. Information on containment zones, hospitals, and hunger relief centers was provided in a geographical context, with directions imparted through Google Maps, a popular user-friendly interface. Regular notifications, worded positively to encourage participation, reminding the user to wash their hands and use masks in public places, were displayed.

### Symptom self assessment

**Figure 3:**
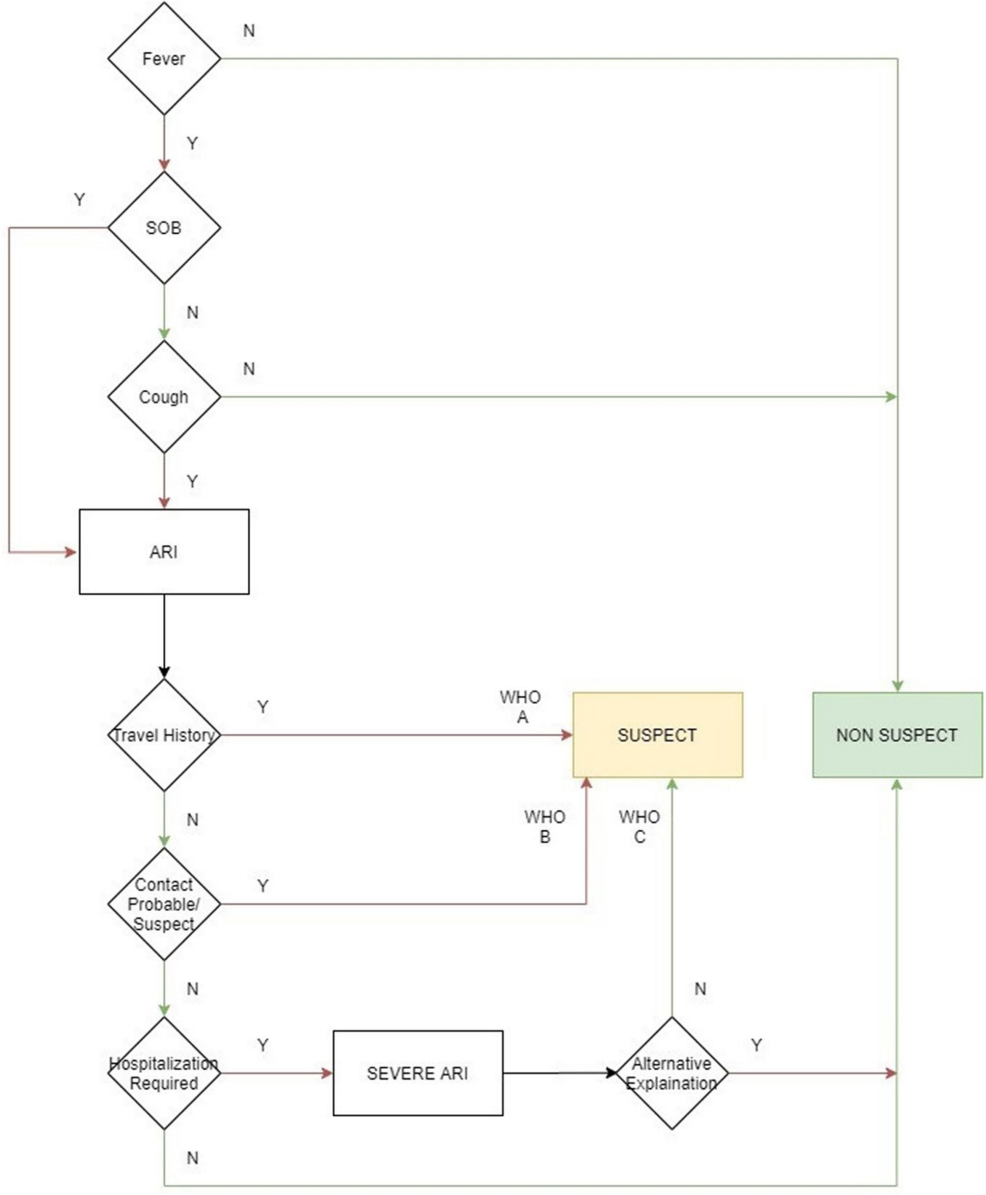
Self assessment tool flowchart. Based on the World Health Organization (WHO) Interim Guidance, a questionnaire and flowchart were developed to classify the responders as ‘Suspects' or ‘Non-suspects'. Here SOB refers to Shortness of Breath, and ARI refers to Acute Respiratory Infection.

Low accessibility of the healthcare system, given the lockdown and social distancing measures in place, and a skewed ratio between the population who wishes to get tested and medical professionals who can verify this need call for an effective alternative to screen patients^18^. Thus, we devised a self-assessment tool for the symptoms of COVID-19, thereby enabling quicker identification of suspect cases who can then be guided to the Government helpline numbers and informed about proper self-quarantine protocols, nearby hospitals admitting COVID suspects, and testing centers. We defined the Suspect Case using the WHO Interim Guidance on Global surveillance for COVID-19 caused by human infection with the COVID-19 virus, and classified them further as a Suspect case (A), (B) or (C)^19^. The 7-point questionnaire was designed using the case definitions from the WHO Interim Guidance verbatim. Based on the WHO criteria’ application on the answers to the 7 questions, the user was notified about whether or not they were suspected of having COVID-19 (Fig. 3).

### Chatbot

**Figure 4:**
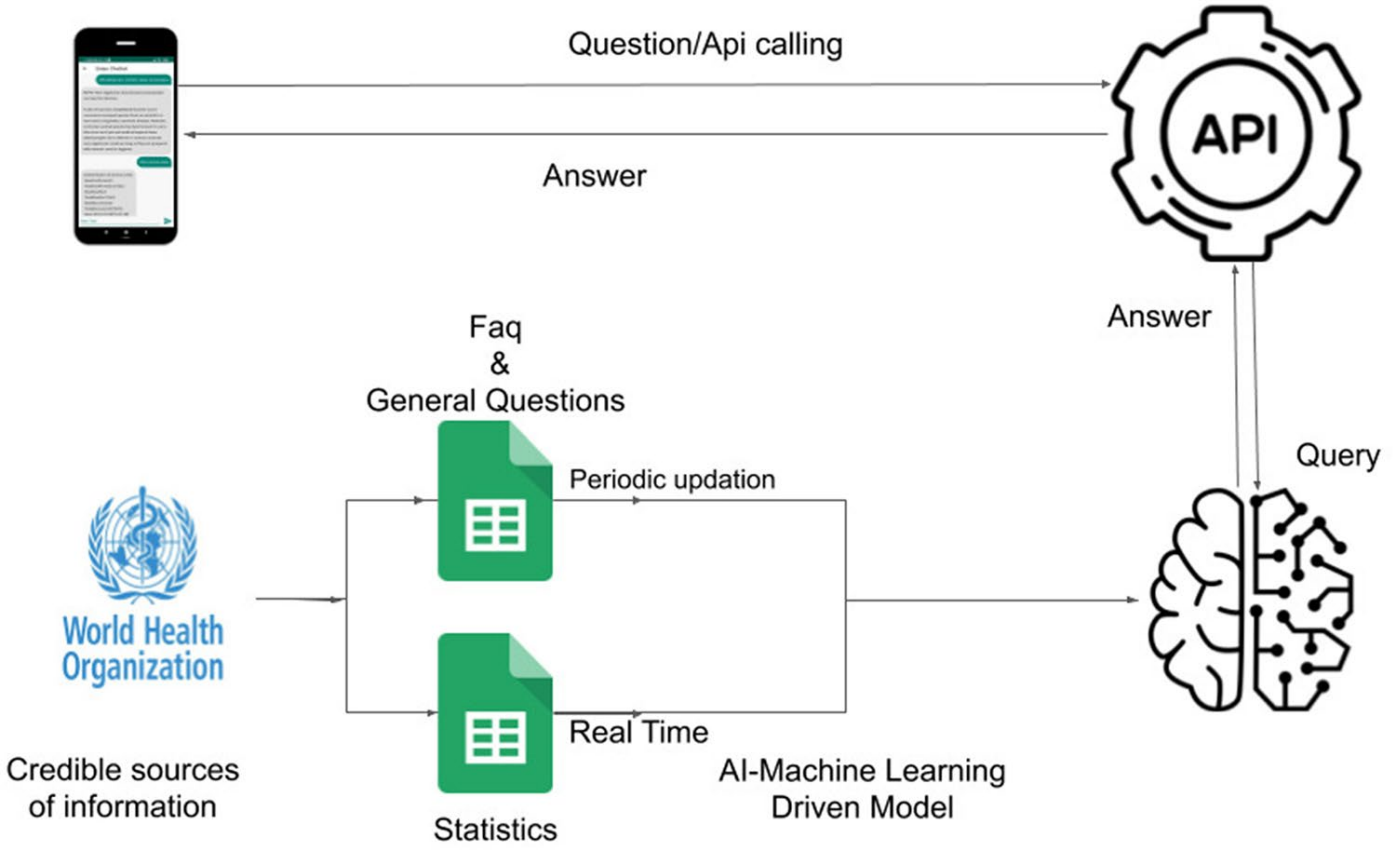
Request-Response cycle in the chatbot. This is a schematic diagram depicting how the answer is displayed whenever a query is asked to the chatbot by a user. This figure was made using **Creately** (URL: https://creately.com/).

Correct and officially verified information regarding the disease should be there at everyone’s disposal. We have made a chatbot (Fig. 4) to serve this purpose, which has verified information from WHO, CDC, and additional government-approved sources. Existing Solution consists of an option Driven System^20^ where a user needs to select through various lists of options to find answers to the Query. Thus, we devised a chatbot system designed to answer user queries using natural language. The current system consists of a Long Short Term Memory (LSTM) model fine-tuned on a Medical Question-Answers Dataset (MedQuAD) dataset^21^. The Dataset was encoded using swivel embeddings generated on the Covid-19 open research dataset^22^. All the data, including the training set, is incubated from Credible and Government controlled sources. Data for Answering the input query was taken from three Sources. Daily Statewise and India case count were extracted and updated on a real-time basis^23^. Data for Training the model was taken from the World Health Organisation's CoronaVirus Frequently Asked Questions^24^ and Centers for Disease Control and Prevention's CoronaVirus Frequently Asked Questions^25^. The user Query is also passed through spelling correction using a symmetric delete spelling correction algorithm along with an artificial increasing frequency of words related to the disease, symptoms, etc.^26^ to increase the accuracy and effectiveness of the system.

### Active user feedback

Anonymized data was collected through self-assessment usage analytics, Play Store managed user statistics, and easy to comprehend survey forms. Our suite deployed an anonymized public health survey that asked basic healthcare-related questions to understand the demographics and monitor the situation periodically. Audio-based feedback was taken from the user to ensure user convenience, establish a two-way dialogue, and prevent specific suggestions from being marginalized. Illustrations were used whenever possible to make the user aware of the collected data, hence protecting their right to information and privacy.

## Results

The study aimed to improve article relevance using a machine learning approach and achieve information dissemination through better engagement in the local language. Our multi-pronged approach targeted to:

(i) Achieve a non-intrusive manner of healthcare knowledge dissemination.
(ii) Use local language to increase the participation of the target group (Female population).
(iii) Develop a self-assessment tool to identify the at-risk population at an early stage to mitigate the chances of community transmission.

### Information enrichment over time: the number of ‘relevant’ votes increases

**Figure 5:**
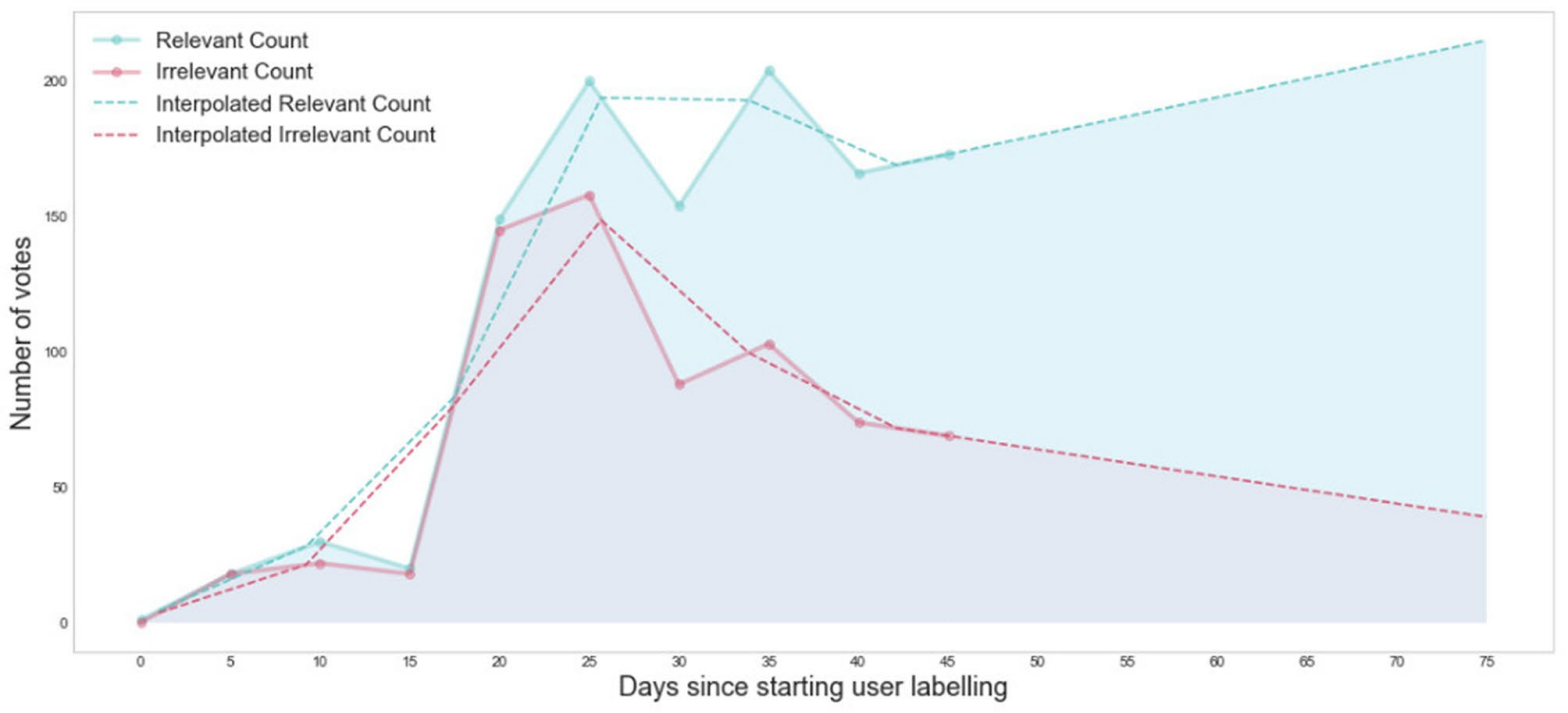
Analysis of natural language processing (NLP) pipeline. The graph shows relevance as a function of user feedback functionality in the app. Relevance is seen to increase with cumulative feedback over time. From day 0 onwards, the Relevant Count’s angular coefficient is 1.39 (± 0.488), the angular coefficient of Irrelevant Count is − 0.99 (± 0.602), with an average slope difference of about 2.29.

With time and increased user feedback, the relevance of matching news articles with WHO reports increases, as seen in the rise in the number of relevant votes, owing to the constantly evolving machine learning model (Fig. 5). The number of irrelevant votes also decreases, validating our proposed methodology and providing increasingly relevant content from trusted sources to the user over time in the language of their preferred choice. At the beginning of the AI-based learning system on 15 March 2020, the number of `relevant' votes and `irrelevant' votes were both 18. On 25 March 2020, with increased user interaction and AI learning, the number of relevant votes was 173 and irrelevant votes was 69. The ratio of relevant votes to irrelevant votes risen from 1.0 to 2.5 over a period of one month.

### Demographics-females engaged more in Hindi

**Figure 6:**
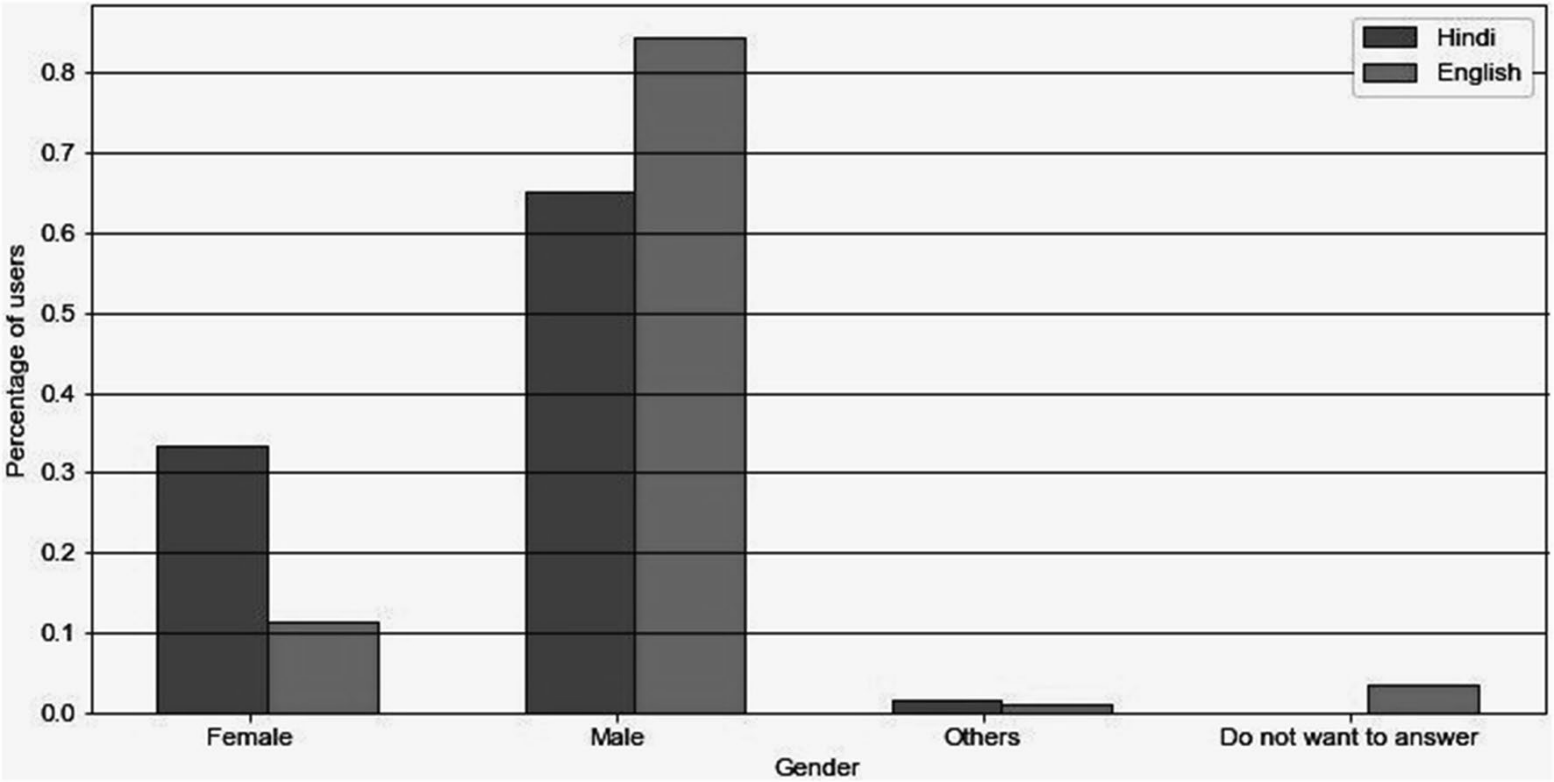
Analysis of public health survey. Distribution graphs showing the distribution of gender among Hindi and English Users. It clearly shows skewness in gender for English users whereas in the case of Hindi users it shows an approximate normalization among the genders.

A total of 436 people took part in the English language based survey, and 126 took part in the Hindi language based survey. Figure 6 was generated based on the survey conducted on the WashKaro app. The analysis of this plot suggests that the number of English users are more than the number of Hindi users. It also depicts that the overall number of male users is more than female users. A key insight observed from the data depicted that Hindi speaking female users (33% of total Hindi speaking users) were more than English speaking female users (11% of total English speaking users). The census of India 2011 highlighted the disparity of literacy rates across genders, with 82.14% literacy rates amongst Indian males and 65.46% literacy rates in Indian females^27^. This underscores the fact that using local languages empowers the sections of the population that might not have otherwise access to the information.

### Target population: users who reported higher than expected incidence

**Figure 7:**
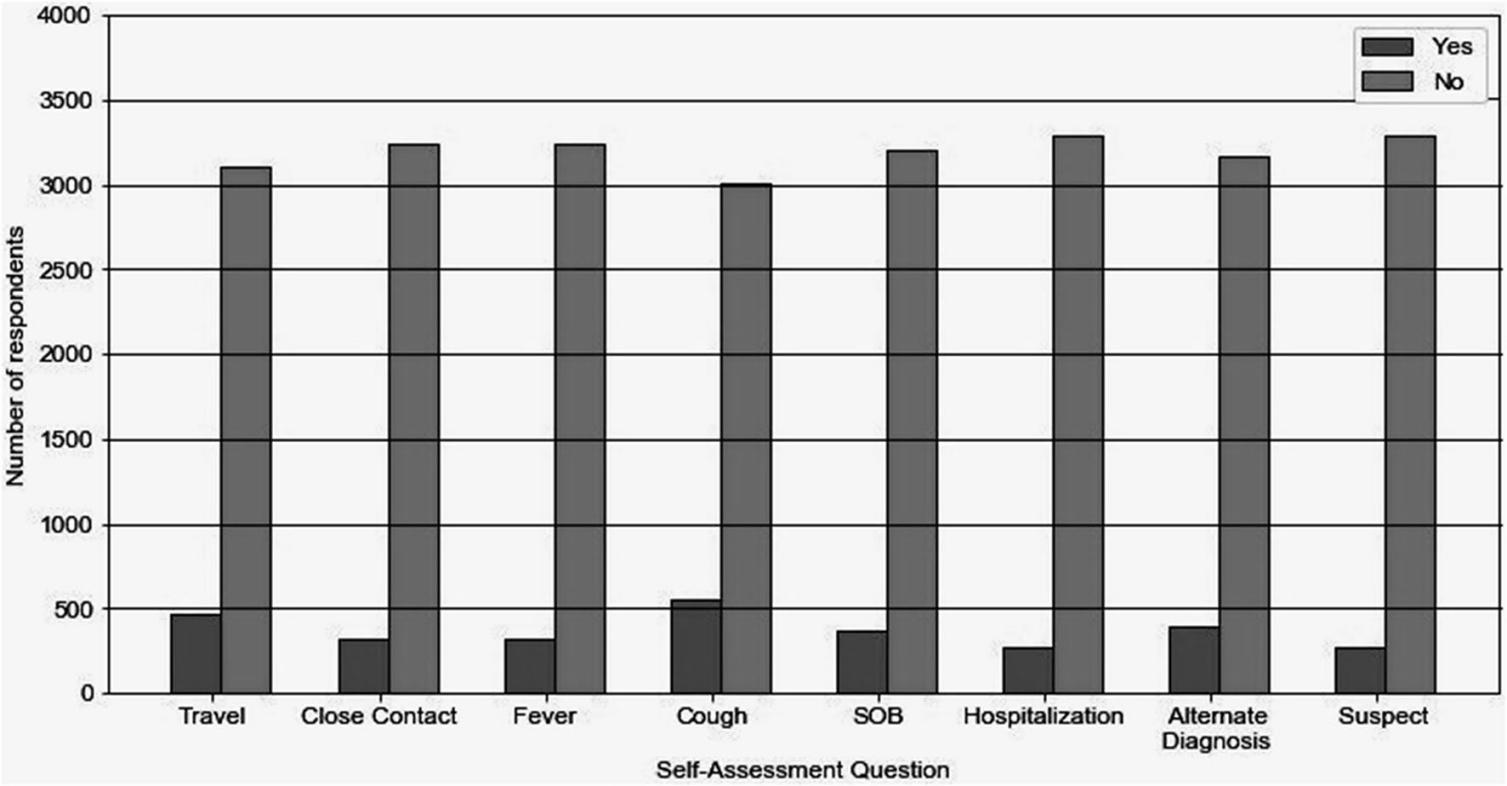
Analysis of self assessment. A simple user-level self-assessment has been deployed to enable the general population to perform self-assessment and identify the population at risk, which can be used as an effective screening. A higher trend for a positive COVID-19 report in people who reported cough was observed. The symptoms of the disease have been known to change with strains, hence this approach of crowdsourcing information provides an agile approach to screen patients with specific symptoms.

Based on the data collected from the symptom self-assessment 276 (7% of 3567 respondents) were found to be suspect cases according to their responses, while 3291 were non-suspect. 467 (13.09%) of the respondents reported a travel history to locations reporting community transmission of COVID-19, 326 (9.13%) reported close contact with COVID-19 positive patient, 323 (9.05% of respondents) reported fever, 556 (15.58%) reported cough, 367 (10.28%) reported shortness of breath (SOB), 277 (7.77%) reported that they required Hospitalization and 395 (11.07%) respondents reported there was an alternate diagnosis for their condition (Fig. 7). A higher trend for a positive COVID-19 report in people who reported cough was observed. The symptoms of the disease have been known to change with strains. Hence this approach of crowdsourcing information provides an agile approach to screen patients with specific symptoms.

## Discussion

Our App was one of the first machine learning and natural language processing based approach to tackle COVID-19 misinformation using machine learning. Over the years mHealth and machine learning have made significant contributions in the medical domain^28^. In the case of COVID-19, where reliable therapeutic strategies are still under experimentation, the role of such mHealth and machine learning interventions is critical^29–31^. Timely dissemination of trusted byte-sized information is extremely instrumental in mitigating the infodemic. With the majority of the world population staying at home due to lockdown, the increased amount of digital consumption opens up the scope to deploy these techniques as an effective social intervention to mitigate the infodemic by delivering the right information to the right people at the right time. When organizations across the globe are proactively testing various strategies to address the issue at hand, open-source software will play a vital role in such scenarios at a global stage to mitigate pandemics and infodemics at the root level.

Our study focuses on addressing these questions and has the following strengths. We provide an open-source template featuring functions like Symptom Self-Assessment, Notification amplifier that notifies the user for washing hands, which are required for fighting against epidemics and pandemics. This also helps in the propagation of the right information hence proper management of infodemic can be done without misleading the masses in crucial scenarios. The enhancement of a few features within the WashKaro application can help in serving as an effective intervention for the government and policymakers. Detailed questions can be formulated targeting the at-risk users identified using symptom self-assessment, which can be incorporated into the existing framework followed by the higher authorities and medical workers for predicting the suspects of COVID-19 at an early stage. After identification and testing of the at-risk population, our analysis can be extended to predict patients who have chances of being at risk in the near future from the definitive set of questions, based on the priority of each question. We can present this data to the suitable administration and decision-makers for taking effective measures against such individuals at an early stage. For the Suspect Cases, we can administer a second questionnaire to further stratify the risk of acute respiratory distress syndrome (ARDS) and septic shock by assessing the severity of symptoms and looking for identified risk factors like age and pre-existing comorbidities that are not included in the WHO Interim Guidance. This can aid in making decisions regarding home quarantine against hospital admission. The app can also be used to identify other suspect cases in the same household. Further, to assist the government authorities to identify those requiring testing for COVID-19, we can ask for contact details of the Suspect Cases with informed consent and relay them to the appropriate government authorities to enable targeted testing. A follow up of the suspect cases through push notifications, advising testing and recording test results, can help ensure that complacence does not set in. Also, as highlighted by previous work, teaching interventions to women can be an important step for mitigating diseases^32^. We reason that the engagement in Hindi is due to the content rather than the nature of the App because the default language of the app content is English. Therefore, the users would have made a conscious effort to change the language to Hindi.

We believe that public health messaging is a key component in managing a health crisis and our approach is geared to make this more agile through machine learning and natural language models. The strength of our approach is in devising a real-world pipeline for local language based deployment of an infodemic mitigation solution. To the best of our knowledge, there are no Applications that use machine learning and natural language processing to provide the right information, to the right people, in the right format, and at the right time. Our pipeline summarized texts from newspapers and matched these to the official sources of information such as the WHO, before delivering these as bite-sized audios in Hindi. The second strength of our study is the online learning algorithm to optimize the threshold of relevance as a function of user feedback. The third strength of our study is in the need for minimum data to gain public health insights. Despite not collecting granular personal data, we were able to show trends such as the gender inequity in the usage of Hindi versus English on the App.

Finally, there are some limitations to the study that have been conducted prior to the revamp of the application. All applications with COVID-19 information were removed in association with the guidelines regarding COVID-19 related applications on the Google Play store^33^. The time frame of the case study for COVID-19 was shortened due to this reason. Our application was the first application providing vetted information using machine learning. As the government rolled out the official apps, these were promoted on a larger scale to target the national audience. That may explain the drop in the number of users active on our app. Secondly, we did not incentivize the responses to the usage of our App. Hence the responses from more than 5000 people are less likely to harbor a systematic bias. The primary objective of this app was straightforward- “Does the user score for relevance increase over time as our natural language processing based filters improve over time with user feedback?” This objective reflects the quality of information and personal preferences such as the language, which were analyzed. This objective is less likely to have heterogeneous influences. Due to data privacy policies and our motivation to collect minimal data, we did not obtain granular information on individuals' locations and other important personal information. Hence the confounding factors may be limited to the number of Android smartphone users (the majority in India) and the rate of spread of disease.

The development of innovative approaches while protecting individual data yet gathering useful inference is an active area of research, and our further work will address this limitation in various public health scenarios. Therefore, we conclude that the role of digital health interventions in the form of systems articulating vetted messages needs to be explored effectively dealing with public health challenges, both during health emergencies and normal times addressing the Sustainable Development Goals (SDGs) put forward by WHO.

Since health information from credible sources is not necessarily prioritized for dissemination in conventional media, especially for less literate and non-English speaking sections of the society. The users were searching for such information at a time when little was available in a local language in India. Our App was the first such platform and > 5000 people downloaded the app during the study window, among those 1545 were actively engaged users before Google pulled the plug on all COVID-19 apps. Going forward, we are continuing to develop natural language processing based services for extending this feasibility experiment for raising public health awareness about infectious diseases such as TB. The further scope and extension of this study involve quizzes, gamification, peer network building and social incentivization to engage users. Future work is planned to evaluate the human-centric design of the app collaboration with an NGO working on the ground. Our use of models to manage information through the use of machine learning optimizes human resources and is shown to be effective as per the measured parameters. We strongly believe that this approach may be relevant to other resource-constrained settings also.

## Supplementary Information

Supplementary Information.

## Abbreviations

WHO: World Health Organization
WASH: Water sanitation hygiene
EPI-WIN: WHO’s information network for epidemics
AI: Artificial intelligence
NLP: Natural language processing
