# Supplementary material for: A machine learning application for raising WASH awareness in the times of COVID-19 pandemic
Source: Sci Rep. 2022 Jan 17;12:810. doi: 10.1038/s41598-021-03869-6 (PMC8764038; doi:10.1038/s41598-021-03869-6)
Supplement: Supplementary file 1 — Supplementary Information. [file 41598_2021_3869_MOESM1_ESM.pdf]

Supplementary

Supplementary section S1: Functionality User Perspective:

WashKaro was the first platform for COVID-related credible health information prioritizing information dissemination in local dialects. Through this application, a user who consumes daily news information would get appropriately matching health guidelines.

1. Users can go to the app's landing page and access various features discussed in the Methods section of the article.
2. Users can click on 'onAir' on the landing page to read/hear a news article.
3. Users can then read/hear the appropriately matched WHO Guideline related to the news article the user has already read.
4. Users can then rate the matching as relevant and irrelevant which improves subsequent matchings. The review from the users was a simple and binary 'tick mark/cross' response. The onboarding of the application clearly stated that the user should "Rate each News and WHO guideline matching" as "Relevant"(Indicated with a tick mark) or "Irrelevant"(Indicated with a cross), so there was little scope for confusion in this user generated feedback.

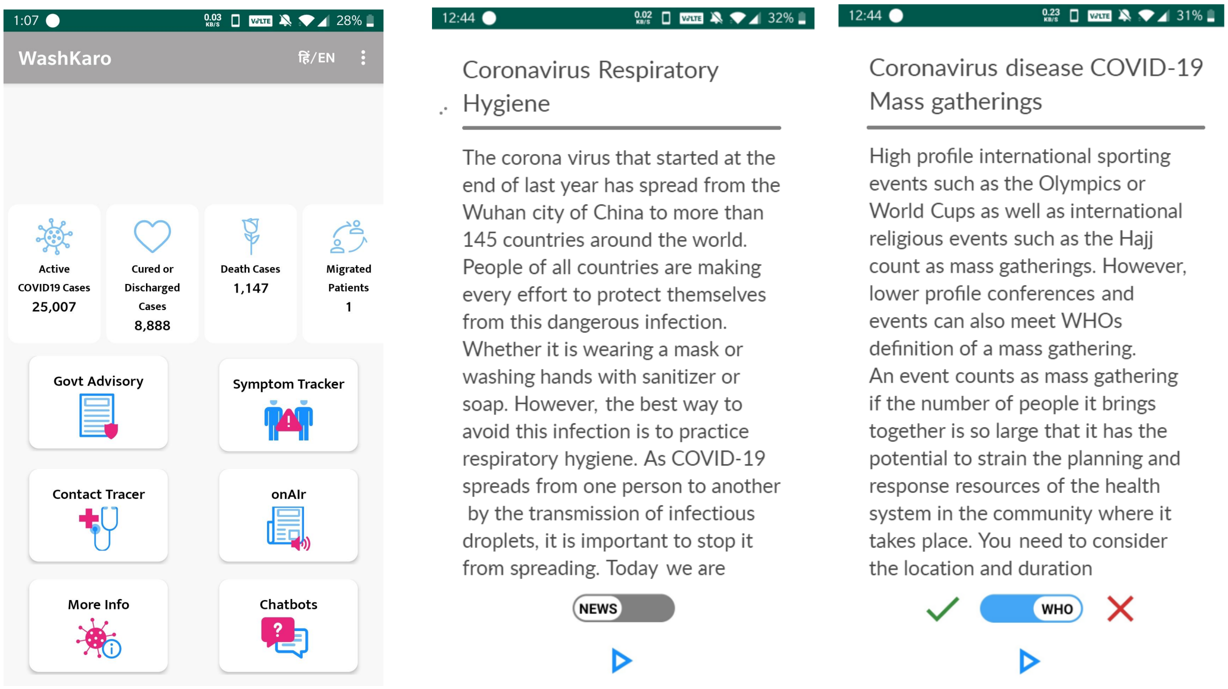

(A) (B) (C)

Supplementary Figure S1: Application screenshots. Figure (A) Shows the landing page of the application. The user can click on onAIr to read news articles. Figure (B) shows the news article

and figure (C) Shows the corresponding WHO guideline matched with the news article. The user can rate the matching as relevant and irrelevant using the icons provided adjacently.

Supplementary section S2: Chatbot

The chatbot used Swivel Embeddings trained on CORD-19 data Data as these mapped relations between commonly used words and scientific terms [1, 2]. These were fine-tuned using TensorFlow 2.x and Keras library on MedQuad Dataset. Text from approximately 2000 answers and Questions from the MedQuad Dataset (equally divided among sections) were fed into the swivel model for fine-tuning. Once the new embeddings were found a Text classifier chatbot was built using the Long short-term memory model. Frequently Asked Questions From CDC and WHO were fed into the classifier. The text classifier model was the best approach due to the lack of data in the early stages of the pandemic instead of the text generator model due to low amounts of training data.

References

1. Noam Shazeer, Ryan Doherty, Colin Evans, Chris Waterson: “Swivel: Improving Embeddings by Noticing What's Missing”, 2016; arXiv:1602.02215.
2. Wang, Lucy Lu et al. “CORD-19: The Covid-19 Open Research Dataset.” ArXiv; 2020. (doi: arXiv:2004.10706)
